# Cardioplegia in Open Heart Surgery: Age Matters

**DOI:** 10.3390/jcm12041698

**Published:** 2023-02-20

**Authors:** Jovana Bradić, Marijana Andjić, Jovana Novaković, Nevena Jeremić, Vladimir Jakovljević

**Affiliations:** 1 Department of Pharmacy, Faculty of Medical Sciences, University of Kragujevac, Svetozara Markovica 69, 34000 Kragujevac, Serbia; 2 Center of Excellence for Redox Balance Research in Cardiovascular and Metabolic Disorders, Svetozara Markovica 69, 34000 Kragujevac, Serbia; 3 1st Moscow State Medical, University IM Sechenov, Trubetskaya 8/2, 119991 Moscow, Russia; 4 Department of Physiology, Faculty of Medical Sciences, University of Kragujevac, Svetozara Markovica 69, 34000 Kragujevac, Serbia; 5 Department of Human Pathology, 1st Moscow State Medical, University IM Sechenov, Trubetskaya 8/2, 119991 Moscow, Russia

**Keywords:** cardioplegia, ischemia–reperfusion, pediatric population

## Abstract

Introduction: Cardioplegia is a pharmacological approach essential for the protection of the heart from ischemia–reperfusion (I–R) injury. Over the years, numerous cardioplegic solutions have been developed, with each cardioplegic approach having its advantages and disadvantages. Cardioplegic solutions can be divided into crystalloid and blood cardioplegic solutions, and an experienced surgeon chooses the type of solution based on the individual needs of patients in order to provide optimal heart protection. Importantly, the pediatric immature myocardium is structurally, physiologically, and metabolically different from the adult heart, and consequently its needs to achieve cardioplegic arrest strongly differ. Therefore, the present review aimed to provide a summary of the cardioplegic solutions available to pediatric patients with a special focus on emphasizing differences in heart injury after various cardioplegic solutions, the dosing strategies, and regimens. Material and methods: The PubMed database was searched using the terms cardioplegia, I–R, and pediatric population, and studies that investigated the influence of cardioplegic strategies on markers of cardiac muscle damage were further analyzed in this review. Conclusions: A large body of evidence suggested more prominent benefits achieved with blood compared to those with crystalloid cardioplegia in pediatric myocardium preservation. However, standardized and uniform protocols have not been established so far, and an experienced surgeon chooses the type of cardioplegia solution based on the individual needs of patients, while the severity of myocardial damage strongly depends on the type and duration of the surgical procedure, overall patient condition, and presence of comorbidities, etc.

## 1. Introduction

Cardiovascular disease (CVD) is a leading cause of death and disability globally, accounting for approximately 17.5 million deaths worldwide every year. The prevalence of CVDs is increasing with age in both genders, while on the other hand, the incidence of congenital heart diseases is reported as 7–9/1000 live births [1,2]. Some CVDs can be addressed with lifestyle changes, medications, or nonsurgical procedures. However, many cardiovascular diseases, such as coronary artery disease and valvular heart disease, require surgical intervention as a part of treatment [1]. Due to a growing need for cardiac surgeries, the total cardiac surgery procedure volume is expected to reach over 1.3 million annual procedures in 2026. During open-heart surgery, it is necessary for the heart to be relaxed and still (non-beating) and for the operative field to be blood-free. The easiest way to achieve a silent operating field is to induce global ischemia to the heart by cross-clamping the aorta, with systemic blood circulation transferred to a heart–lung machine [3,4]. Although convenient for the surgeon, ischemia that occurs during the procedure is detrimental and can be tolerated for only a few minutes without additional protection [4].

The absence of oxygen and metabolic substrates for cardiomyocytes occurring during ischemia can cause functional, structural, and metabolic changes [5]. During ischemia, myocardial cells switch from aerobic to anaerobic metabolism resulting in the accumulation of lactate and the generation of acidosis, leading to a decrease in intracellular pH, while myocardial contraction is impaired due to alterations in calcium ion homeostasis [6,7,8]. As ischemia is a very progressive process, the longer the ischemic duration, the more severe the changes in cells and molecules, eventually leading to cell death without timely reperfusion [3,8]. Although the restoration of blood (reperfusion) to an ischemic heart is essential in order to save the myocardium, it can paradoxically cause irreversible myocardial damage termed “ischemia–reperfusion (I–R) injury”. Reperfusion after an ischemic episode may result in cardiomyocyte dysfunction caused by the production of reactive oxygen species (ROS), intracellular and mitochondrial Ca^2+^ overload, and the accumulation of inflammatory cells [5,9,10].

In order to minimize I–R injury and avoid the mentioned consequences, several myocardial protective methods were introduced. The primary heart-protection method used during both adult and pediatric cardiac surgery is cardioplegia, a pharmacological therapy administered in order to intentionally and temporarily arrest the heart. During congenital heart defect reparations, it is of a crucial importance to provide cardioplegic solutions along with hypothermia; however, myocardial injury routinely occurs in pediatric surgery and might lead to a fatal outcomes. Interestingly, heterogeneity in cardioplegia practices exists in medical institutions worldwide, which certainly affects the clinical outcomes in children. Data suggest that blood cardioplegia is the most commonly used; however, the choice of the type of solution, temperature, and dosing intervals differ among surgeons [5].

Taking the aforementioned facts into consideration, the aim of the current review was to summarize the available data referring to cardiologic protocols implemented in pediatric heart surgery worldwide, reveal the current practice, and highlight the impact of adjuncts in solution on the extent of myocardial injury. Studies that investigated the influence of cardioplegic strategies on markers of cardiac muscle damage, such as cardiac troponin I (cTnI), troponin T (cTnT), and creatine kinase MB isoenzyme (CK-MB), were further analyzed in this review. On the other hand, studies that only followed effects based on clinical outcomes were not the focus of this paper.

## 2. Data Sources

We searched the PubMed database using the terms cardioplegia, ischemia/reperfusion, and pediatric population. Studies that investigated the influence of cardioplegic strategies on markers of cardiac muscle damage, such as cTnI, cTnT and CK-MB, were further analyzed in this review. Titles and abstracts were screened, and relevant full-text articles published in English to December 2022 were included.

## 3. Differences in the Response of Adults and Pediatric Hearts to I–R

**Figure 1:**
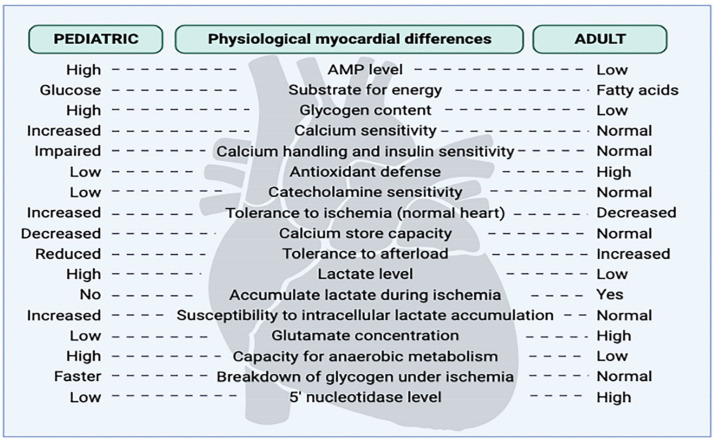
Differences in the pediatric and adult myocardium.

As the pediatric immature myocardium is structurally, physiologically, and metabolically different from the adult heart, their needs to achieve cardioplegic arrest strongly differs (Figure 1) [11]. There is a growing body of evidence that the myocardium of newborns and infants is more tolerant to ischemia than the heart of an adult [12]. However, so far, the mechanisms that explain age-related differences in myocardial recovery have not been clearly defined. Several differences, which can better influence the immature heart’s ability to tolerate ischemia, are reflected by higher preischemic levels of tissue glycogen stores, resistance to postischemic intracellular calcium and sodium accumulation, altered high-energy phosphate catabolism during ischemia and reperfusion, increased ability to use glycolysis during ischemia, and better H^+^ buffering capacity [13]. Despite a large number of studies aimed to examine safe and effective myocardial protection, the different needs of an immature and mature myocardium and their response to ischemia remain contradictory. Therefore, the purpose of this review article is to summarize and indicate potential differences in the choice of cardioplegia, the dosing strategies, and variable clinical outcomes in neonates and adults. Great scientific attention has been focused on discovering the optimal therapeutic protocol for pediatric immature heart preservation. However, the best therapeutic protocol has not been established yet. Currently, the cardioplegic strategy consists of adjusting the cardioplegic content and protocol to the demands of the pediatric myocardium [11,14].

Even though myocardial protection is essential for successful cardiac surgery, the most effective choice for cardioplegia remains unknown, and the search for an ideal cardioplegic solution is ongoing. Therefore, the present review aims to summarize the effects of cardioplegic solutions on myocardial protection in order to provide a better understanding how cardioplegic strategies should be tailored to different ages and pathologies.

## 4. Cardioplegia as a Tool for Heart Preservation throughout History

In the late 19th century, scientists discovered that a high concentration of potassium could stop the heart in diastole, which could be used as an approach in open-heart surgery. However, refractory ventricular fibrillation following the procedure raised a concern and limited the concept of high-serum potassium cardiac arrest. In the early 1950s, doctor Melrose continued research in this field by formulating a potassium citrate solution since he hypothesized that cardiac complications previously described are derived from chloride ions but not from potassium. He noticed that high concentrations of potassium citrate, applied via infusion into coronary arteries, caused reversible cardiac arrest. The concentration of the applied potassium citrate solution was 77 mmol/L. The mechanism that can explain cardiac arrest involves depolarization of the myocardial membrane, i.e., the influx of calcium ions leads to contraction, while the release and sequestration of calcium causes heart arrest in the diastole phase [15]. The most important finding is that after cardiac arrest, the heart recovered its function without myocardial injury, so this strategy was introduced to cardiac surgery as the “Melrose Technique” with the potential to provide a bloodless field for surgical maneuvers with the preservation of heart function [16,17]. Nevertheless, further examinations suggested that this type of hyperkalemic solution still predisposed the heart to contractile dysfunction, ventricular fibrillation, and consequently cardiomyocyte death. These post-operative complications limited clinical usage and led to the abandonment of hyperkalemic solutions for more than a decade [18]. Afterwards, there have been several attempts to replace potassium with ischemic stasis caused by aortic occlusion, topical hypothermia, etc.; however, they did not prove to be superior to the aforementioned hyperkalemic solution [19,20]. Initially, heart dysfunction was thought to be due to the presence of anionic salts, but in 1975, Tyers et al. refuted this hypothesis and discovered that complications with the “Melrose technique“ were due to the high concentration of potassium ions [21].

The end of 1960s and beginning of 1970s was associated with the return of a high-to-moderate potassium concentration solution as an attractive strategy in triggering cardioprotection. In fact, in 1975 St. Thomas’ Hospital (STH) solution was introduced by Hearse and Braimbridge in open-heart surgery in London. STH represents one of the widely used extracellular cardioplegic solution worldwide [22]. Investigations were continued with the aim to discover how the composition of this solution could be modified in order to improve the protection of the heart. As a result, several years later, an improved formulation of this solution, known as modified STH solution or STH solution No. 2 (STH 2), was developed. While both types of solutions contained increased magnesium (16 mmol/L) and normal calcium ion concentrations, they differed in potassium ion concentrations. The first STH contained potassium ions at a concentration of 20 mmol/L, while the second one contained a lower content, i.e., 16 mmol/L. The mechanism responsible for the effects of these solutions is based on diastolic arrest via membrane depolarization [23].

**Figure 2:**
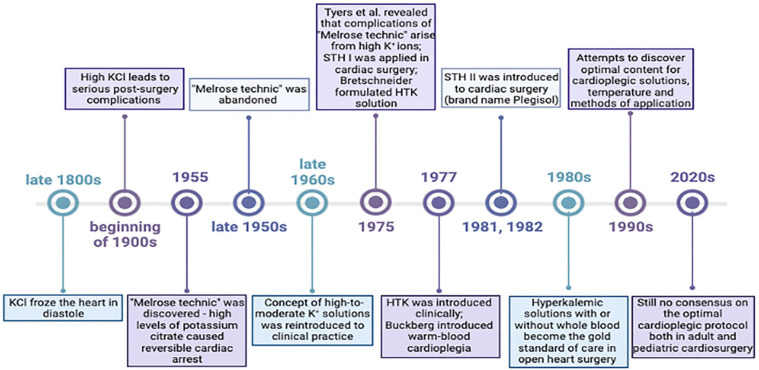
Cardioplegia as a tool for heart preservation throughout history.

In 1970s, Bretschneider and his group formulated Histidine-Tryptophan-Ketoglutarate (HTK) solution as an intracellular crystalloid solution for administration in cardiac surgery. Moreover, this solution was introduced into clinical practice in 1977 and modified in the 1980s [24,25]. Although HTK solution was primarily intended for use in cardiac surgery, it has found a place in the preservation of other organs, such as the liver, pancreas, and kidneys. Nowadays, after subsequent modifications, it is available as Custodiol^®^ and has been extensively applied as a cardioplegic strategy for myocardial protection [26]. In the following years, additional research was conducted, which led to the development of several cardioplegic solutions that differ in the composition, method of application, and temperature of the solution used (Figure 2).

### 4.1. Membrane Polarity as a Target for Cardioprotection

#### 4.1.1. Depolarized Arrest

**Figure 3:**
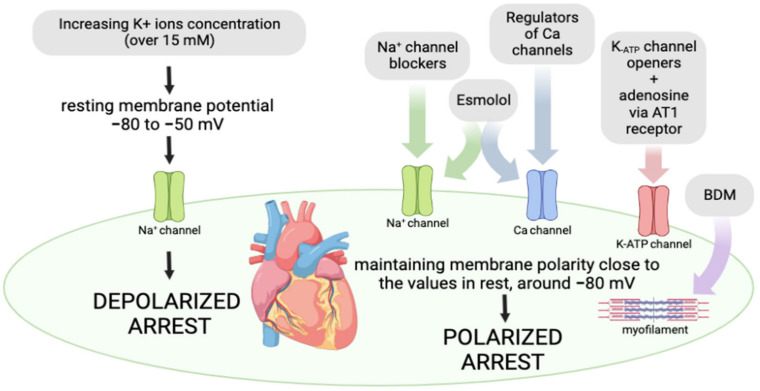
Targets for achieving depolarized and polarized arrest.

It has been well known that intracellular potassium levels play a pivotal role in the maintenance of cardiovascular system homeostasis. Physiological potassium levels below 16 mM produce a resting membrane potential of approximately −85 mV, thus enabling the hyperpolarization of smooth muscles in blood vessels. Nevertheless, increasing potassium ion concentrations (16 mM–25 mM) elevate the resting membrane potential to a less negative value compared to baseline values, i.e., to approximately −80 mV to −50 Mv [18,27]. At these values of resting membrane potential, cardiomyocytes promote the inactivation of fast voltage-dependent sodium channels, thus preventing phase 0 of myocardial action potential. Additionally, in this depolarized state, Na^+^ ions can enter across the small Na^+^ “window” current, thus causing an elevation in intracellular sodium ion levels, which promotes a reversal of the voltage-dependent Na^+^/Ca^2+^ exchanger. As a result, there is an exchange of three sodium ions for one calcium ion, thus enabling intracellular calcium overload. Due to ionic homeostasis alterations, cardiomyocyte contractility and excitability are disturbed, resulting in diastolic arrest. Nowadays, most cardioplegic solutions contain potassium at a concentration of more than 15 mM and represent the standard-of-care for cardioplegic arrest in open-heart surgery [27], (Figure 3).

#### 4.1.2. Polarized Arrest

Although depolarized cardiac arrest as an approach has been widely used in cardiac surgery, the occurrence of myocardial stunning, arrhythmias, and inflammation, etc. have led researchers to look for a different strategy for providing myocardial protection. In that sense, hyperpolarization has been imposed as a subject of interest to scientists as a potentially promising alternative to depolarized cardioplegia [18]. Hyperpolarization represents one popular avenue of investigation with a greater capability to preserve myocardial function and structure. A strategy of ‘’polarized arrest’’ is based on the application of pharmacological agents that maintain the polarity of the membrane close to the potential at rest. These compounds appear to be superior in comparison to hyperkalemic solutions due to a capacity to decrease energy consumption and metabolic demands of the heart since the calcium channels are not activated at these values of membrane polarity [28,29]. The most frequently used polarized agents involve Na^+^-channel blockers, ATP-sensitive K^+^ (K-ATP) channel openers, calcium antagonists, and esmolol, etc. Na^+^-channel blockers, such as lidocaine, procaine, and tetrodotoxin (TTX), act by blocking the voltage-gated sodium fast channels, thus preventing the 0 phase, i.e., fast depolarization in the cardiac cycle. The activation of K-ATP channels by nicorandil, diazoxide, and adenosine during reduced blood and oxygen supply to the heart leads to a drop in the duration of action potential, thus minimizing the deleterious effects of ischemia on the myocardium. A drop in the duration of action potential might decrease the influx of Ca^2+^ via L-type channels and release it from sarcoplasmic reticulum, which strongly influences cardiac contractility [30]. Protocols for the application of cardioplegia are not uniform and differ according to the type of solution used, the temperature of the cardioplegia, the method of perfusion, and the duration of perfusion (Figure 3).

#### 4.1.3. Routes of Cardioplegic Solution Application: Advantages and Disadvantages

The cardioplegic solution is applied to the coronary arteries and causes electromechanical cardiac arrest, which gives cardiac surgeons the opportunity to perform surgery in a bloodless field. The administration of cardioplegia can be anterograde, retrograde, or both. Anterograde cardioplegia involves an application that follows anatomical routes and regular coronary circulation through insertion in the proximal aorta. On the other hand, retrograde perfusion is performed by direct intubation of the coronary sinus, and it is indicated in patients with severe aortic regurgitation and severe coronary artery stenosis. Retrograde perfusion is suggested for patients with a hypertrophic myocardium and severe coronary heart disease because anterograde applications will not provide adequate myocardial perfusion [15,31,32,33,34].

#### 4.1.4. Different Temperatures of Cardioplegic Solutions: Advantages and Disadvantages

Cardioplegia can be performed with hot, cold, and intermediate lukewarm solutions. The most common application in clinical practice involves the application of cardioplegic solutions together with hypothermia, because lowering the temperature also reduces the energy needs of cells. The application of cold cardioplegia takes place for about twenty minutes in order to maintain the heart temperature in the range of 10–15 °C. It has been shown that in this way, the heart can be exposed to ischemia for about 4 h [35]. Hypothermia with cardioplegia reduces the needs of the heart by about 5%. The current gold standard of cardiac myocardial surgery is the use of cardioplegia, while the optimal temperature of the cardioplegia solution is a matter of debate. Early cardioplegic methods used cold crystalloid solutions to induce and maintain cardiac arrest during heart surgery. Although hypothermia has the advantage of reducing the heart’s need for oxygen, the question arises as to what extent it is harmful due to disruption of myocardial homeostatic processes. Hypothermia can impair enzyme activity, elicit cell membrane instability, and impair glucose utilization and ATP production [36,37]. Warm-blood cardioplegia was introduced into clinical practice in 1977 and has been shown to improve myocardial recovery and oxygen delivery, reduce intercellular edema, and stabilize the membrane. Dangerous complications of warm blood cardioplegia are perioperative strokes and neurological injuries due to the sensitivity of the brain to normothermia. Warm or intermediate lukewarm heart surgery often leads to vasodilation on the cardiopulmonary bypass, which requires the use of alpha agonists due to the reduced perfusion pressure. It is believed that the disadvantages of the described cardioplegia temperatures can be overcome by applying warm blood cardioplegia in the onset of cardioplegia, with the continuation of cold blood cardioplegia in the maintenance phase [36,37,38,39,40].

#### 4.1.5. Continuous and Intermittent Cardioplegia: Advantages and Disadvantages

Continuous cardioplegia possess certain advantages over multidose cardioplegia in the case of clogged blood vessels. Namely, continuous application better supplies the myocardium with a solution and largely removes harmful metabolites [41]. However, continuous blood anterograde cardioplegia interferes with the visibility of the operative field, so there are requirements for interrupting it for fifteen minutes from time to time to ensure visualization of the field. While continuous use is characteristic of normothermic cardioplegia, intermittent use is associated with cold cardioplegia. Solution del Nido and Custodiol appear to be suitable solutions because they are applied in a single dose that allows an effective duration of cardioplegia of up to 90 and 180 min [38,42].

#### 4.1.6. Type of Cardioplegic Solutions

Over the years, various cardioplegic solutions and methods have been developed, with each cardioplegic approach having its advantages and disadvantages. All of them should be used appropriately, taking into account the characteristics of myocardial injury and the duration of ischemia. The ideal cardioplegic solution should provide safe and permanent rest of the heart during the intervention, while reducing the change in electrolytes through the myocardial cell membrane. Cardioplegic solutions can be divided according to their composition into crystalloid and blood cardioplegic solutions. An experienced surgeon chooses a cardioplegic solution based on the individual needs of patients in order to provide optimal heart protection while reducing complications.

##### Crystalloid Cardioplegia

Crystalloid cardioplegia has been implemented in clinical practice for more than four decades [43]. Based on the composition of electrolytes and the consequent differences in the mechanism of cardiac arrest, crystalloid solutions can be classified into two types: extracellular, which contains high concentrations of Na^+^, Ca^2+^, and Mg^2+^, and intracellular, which contains low concentrations of Na^+^ and Ca^2+^ (Table 1). While extracellular solutions induce cardiac arrest via depolarization, intracellular solutions reduce the membrane potential resting state, thus preventing the generation of action potentials. Even during prolonged ischemic challenges encountered during heart transplantation, it is necessary to collect the results of a large number of patients in order to discover and compare the effectiveness of intracellular versus extracellular solutions. Cold crystalloid cardioplegia can be administered anterogradely or retrogradely, periodically for about twenty minutes.

Crystalloid solutions that have been most commonly used in pediatric surgery include STH and HTK (Custodiol) solutions. The detailed characteristics of each of these solutions are described below, but common to all crystalloid cardioplegic solutions is a K^+^ content between 10 and 40 mmol/L (mEq/L) and the common addition of bicarbonate [31,35,44].

##### Most Commonly Used Crystalloid Cardioplegia in the Pediatric Population

STH solutions. A solution that has been used in practice for the longest time is STH, which represents an extracellular crystalloid cardioplegia. Procaine is added to the solution in order to reduce the need for oxygen and improve heart recovery. In a previous study, the effect of adding procaine in increasing concentrations to the modified STH solution was assessed, and it has been shown that a concentration of 0.05 mmol/L leads to an improvement in myocardial recovery by 15–20%, which indicates its positive effect in cardiac surgery. If the concentration of procaine is increased slightly, no protective effect would be observed, while with a further increase, harmful effects could be observed [23].

HTK (Custodiol). Also known as HTK (histidine–tryptophan–ketoglutarate) solution, it is an intracellular crystalloid solution that has been also used in cardiac surgery. Commercial Custodiol solution contains a low concentration of sodium for performing cardiac arrest in diastole by inhibiting the rapid phase of the action potential [45]. This solution also contains histidine, which is a buffer, tryptophan, which has a role in stabilizing the cell membrane, and ketoglutarate, which is supposed to promote the production of ATP during reperfusion [46]. In addition, Custodiol solution contains mannitol, which helps in reducing cellular edema and eliminating pro-oxidans. The use of histidine as a protein buffer instead of bicarbonate can have several advantages in order to maintain the intracellular pH. Nowadays, a high concentration of histidine, such as 198 mM, in HTK solution is routinely used to protect the myocardium in cardiac surgery [47]. Custodiol is formulated in order to achieve heart protection for 4 h after one administration, so it is attractive to cardiac surgeons because it enables the correct technical flow of surgical procedures without interruption. Adequate cardioprotection is reached thanks to the selected components of the solution.

##### Blood Cardioplegia

Crystalloid cardioplegia was present until the early 1980s, after which the author Buckberg showed that the addition of blood as an agent for transmitting cardioplegia solutions could have numerous benefits. Buckberg et al. introduced the concept of applying blood solutions that have advantages over crystalloids [48,49]. Namely, the addition of blood achieves improved oxygen delivery and provides ideal oncotic pressure, as well as the efficient maintenance of pH through blood buffer. Moreover, in that period, the same author and his co-workers pointed out that changes in the composition of reperfusions, temperature, and pressure can alleviate the damage caused by reperfusion [50]. The advantage of intermittent over continuous application of these solutions is clearly shown. Blood cardioplegia today involves solutions that contain one part crystalloid solution and one part blood or one part crystalloid solution and four or eight parts blood. In current clinical practice, standard blood cardioplegia has been widely used [51].

Del Nido cardioplegia has been used worldwide for patients of all ages, from neonates to adults, and the content is presented in Table 2. The base solution is Plasma-Lyte A which represents a crystalloid component with a content similar to extracellular fluids, without calcium. In order to obtain del Nido solution, this crystalloid part is mixed with blood to achieve the ratio of four parts of crystalloid to one part of blood. Hyperosmotic mannitol helps in reducing myocardial edema, sodium bicarbonate helps in maintaining intracellular pH, and lidocaine contributes to polarizing the cell membrane, thus counteracting the adverse impact of hyperkalemic depolarized arrest on the myocardium [52,53].

## 5. How Adjuncts in Cardioplegic Solutions Affect the Extent of Myocardial Injury in the Pediatric Population: Data from Clinical Studies

Despite numerous studies conducted in the pediatric population, it is still not possible to clearly identify the best and most effective protocol in all children. In the following text, there is a review of studies that investigated the effects of blood and crystalloid cardioplegia, as well as the influence of factors, such as age and cyanosis, in children on the extent of myocardial ischemic injury (Table 3). Studies that monitored the effects of cardioplegia on cTnT, cTnI, and CK-MB were analyzed in this research, while the studies that only followed time spent in the intensive care unit and necessity of inotropic support, etc., without markers of myocardial damage were not included.

The undoubted advantages of crystalloid solutions involve the long-lasting effect of a single dose, easier application, and lower cost. Additionally, the standardization of crystalloid cardioplegia among different health care institutions is simpler compared to blood cardioplegia. In fact, the complexity of the standardization of blood cardioplegia is a consequence of a variety of available delivery techniques. Crystaloid solutions do not alter the visibility of the operative field as blood cardioplegia does, which is of a great significance since enabling a visible operative field is one of the most important facts for performing surgical maneuvers [51].

In 1997, the first clinical trial was conducted with the focus on addressing the question of the effectiveness of blood cardioplegia compared to crystalloid solutions in a pediatric population. This research involved 138 pediatric patients and revealed that both types of cardioplegia solutions, applied with an antegrade hypothermic dosing strategy, improved the postoperative ventricular function. Interestingly, blood cardioplegia did not exert protective effects in cyanotic patients [54].

In the early twenty-first century, information regarding cardioprotection in heart surgery was scarce since strategies proven in adults were implemented in pediatric surgery without enough investigation and adjustments to the needs of pediatric hearts. A study conducted during the beginning of 21st century focused on the influence of crystalloid cardioplegia but in different age groups. It included fifty-eight patients randomly divided into a group of infants and children over 12 months of age who were subjected to elective open heart surgery. It was discovered for the first time that factors, such as age and degree of cyanosis, influence metabolic alterations in ischemic–reperfusion conditions. This group of authors showed that crystalloid cardioplegia is associated with myocardial injury with different heart responses in reperfusion depending on the age and occurrence of cyanosis [55]. Cyanosis was associated with worse clinical outcomes in children, which could be explained by impaired calcium handling together with metabolic demand, while it did not affect heart functional recovery in infants [56]. Although it has been well established that lactate levels are elevated in ischemic conditions, this is not the case for the myocardium of infants and cyanotic children, indicating that anaerobic metabolism in ischemia differs in these hearts compared to those in acyanotic children. Lactates in the myocardium of acyanotic children may serve as a substrate for energy production and can protect the heart by decreasing the intracellular pH value [57]. The limitation of this study was the absence of results observed in neonates, as well as in children with an acute high degree of cyanosis [55].

Further research in this area, which focused on the differences in cold blood and cold crystalloid cardioplegia, was conducted by Caputo and his coworkers [58]. Forty noncyanotic children were randomly assigned to receive one of the aforementioned protocols for myocardial protection. This research did not reveal different influences between cold blood and cold crystalloid cardioplegia on clinical outcomes; however, they noticed lower metabolic disturbances related to the blood cardioplegic solution. It was noticed that the response to crystalloid (St Thomas I) solution differs in infants and children. In fact, a more prominent decrease in intracellular ATP and the higher release of cTnI were detected in infants, suggesting less resistance to ischemia in this age population. Interestingly, there was no difference in the myocardial functions of infants and children when receiving blood cardioplegia, which may be attributed to blood buffer potential and antioxidant properties. Generally viewed, more pronounced benefits of blood cardioplegia would be expected if the myocardium would be subjected to a longer period of ischemia. A limitation of this study included the study population subjected to only ventricular septal defect repair with a short cross-clamp time.

Two years later, Modi et al., in 2004, compared three different approaches, cold crystalloid, cold blood, and cold blood cardioplegia, during ischemia followed by a warm blood solution in reperfusion in children [59]. One hundred and three children were recruited into the study, and right ventricular biopsy samples were collected, cellular metabolites were assessed, and postoperative serum cTnI levels were determined. It is important to note that the STH crystalloid solution was associated with more pronounced cardiac damage in hypoxic hearts and was not sufficient to preserve the heart function, while blood cardioplegia had the capability of protecting the myocardium from more severe injury. In this study, a novel combination of cold and warm cardioplegia appeared to provide the most powerful cardioprotection. It is assumed that the benefits of the introduction of warm blood cardioplegia in reperfusion are precise in terms of improving the metabolic status and attenuating myocardial stunning linked to crystalloid cardioplegia [60]. Higher glutamate levels in cyanotic patients receiving cold blood cardioplegia for surgery and terminal warm blood cardioplegia in reperfusion may explain the recovery of the ischemic heart and regeneration of energy. Another study also confirmed the advantages of blood over crystalloid cardioplegia in terms of better myocardial function and metabolism preservation [61].

One cohort study involved neonates and infants subjected to biventricular repair surgery. One group of children were receiving Plegisol, while others were managed with a mix of crystalloid cardioplegia and dilute blood (four parts of crystalloid to one part blood). The cardiac index was determined and the postoperative period was monitored. Plegisol exerted better cardioprotective effects in neonates, while this advantage appeared to be less pronounced with age [62]. A further examination was focused on differences between a modified adult multidose solution and the del Nido single-dose solution, and it was found that Del Nido solution was safe and the efficacy was similar or even better compared to that of the modified adult multidose solution [63]. The great benefit of del Nido was based on the possibility to apply only one dose of solution for over 90 min of the aortic clamp period [64].

In 2013, a group of researchers decided to investigate and compare for the first time repeated oxygenated warm blood cardioplegic solution and Custodiol in a population of 218 neonates [65]. Until 2013, there was only information regarding a comparative analysis of Custodiol and cold blood cardioplegia in animal studies with controversial findings. The protocol for complex procedures with long cross-clamping durations has not been precisely established and it was reported that repeated WBC can be effective in pediatric patients and neonates [64,65]. Oxygenated WBC was associated with a drop in cTnI release and a shorter stay in intensive care units, thus suggesting better protection with WBC. In the study conducted by Qulisya et al. in 2016, the greater potential of blood cardioplegia applied at a temperature of 10–15 °C in preserving the myocardium was also observed in children compared to that of Custodiol. The higher incidence of adverse outcomes in children receiving Custodiol might be explained by the longer time of mechanical ventilation and more frequent low cardiac output syndrome [66]. Another study suggested slight differences between blood and Custodiol cardioplegia, which did not affect the postoperative clinical outcome [67].

Although DN cardioplegia was firstly introduced at 1990s, the first extensive study aimed at comparing its efficacy and safety with those of a standard cold blood cardioplegic solution was conducted in 2017. DN solutions are interesting due to their convenient application in a single dose that does not require interrupting the procedure for repeating the dose and prolonging aortic cross-clamp times as in case of STH solutions. This prospective randomized clinical trial strongly suggests the superior effects of del Nido cardioplegia compared to those of conventional cold blood cardioplegia (STH) manifested as long-acting effects, better impact on both cardiac function and structure, and a reduction in mortality [68]. In accordance with this finding, later research also confirmed slightly better cardioprotection after the administration of del Nido solution in a pediatric population compared to that with conventional blood cardioplegia [69]. The better effects of del Nido solution may be attributed to the presence of lidocaine, which decreases the risk for arrhythmias and prevents intracellular Ca^2+^ accumulation and consequently cell injury [70,71]. Another protective feature of del Nido solutions involves the capacity to promote this anaerobic glycolysis and decrease energy consumption, while del Nido solution is also characterized by low hematocrit and therefore requires the addition of mannitol to decrease myocardial edema [72]. It may be concluded that the highest benefits of del Nido solutions can be achieved in complex cardiac surgeries that require long aortic cross-clamp times, while in simple procedures with shorter aortic cross-clamp times, the difference is less pronounced.

Evidence suggests that one dose of del Nido is sufficient for an aortic cross-clamp duration of over 90 min, while a longer period would require an additional dose. Previous research suggests shorter aortic cross-clamp times, as well as reduced or similar levels of markers of myocardial injury, after del Nido compared to those in blood cardioplegia pediatric patients [66,73,74]. A recent cohort study from 2022 also confirmed the advantage of del Nido solution over ST blood cardioplegia in terms of the decrease in the aortic cross-clamp time and number of doses, but in neonates [75]. It should be highlighted that most studies proving the advantages of del Nido compared to blood cardioplegia were conducted on patients with a short cross-clamp period, while there is a lack of comparative analyses of these two cardioplegic strategies in patients subjected to prolonged ischemia. There is still a question of whether blood cardioplegia is efficient only in hypoxic hearts, and the precise role of blood-based cardioplegia in the protection of the immature myocardium remains unclear.

When the efficacy of STH and del Nido cardioplegia was assessed in a randomized controlled trial in a pediatric population, no difference in myocardial injury, i.e., the level of cTn-I, was revealed, while STH appeared to be more potent in reducing the myocardial inflammatory response to ischemia and improving metabolic status [76].

Importantly, hemodilution as a complication following cardiopulmonary bypass leads to increased hospital stays and mortality rates. Therefore, it is important to choose the type of cardioplegic solution associated with a lower risk of hemodilution, such as blood compared to crystalloid cardioplegia [77,78].

**Table 3:** Clinical studies in pediatric surgery in which markers of myocardial injury were monitored.

Surgical Procedure	Patients Age	Applied Cardioplegia	Dosage and Administration	Temperature	Patients Per Group	Parameters of MI Assessment	Conclusion. Study/Year
Open cardiac surgery	1–120 months	STH I	Anterograde administrations of 25 mL/kg/min for 4 min	4–6 °C	27 infants	cTnI	STH cardioplegia is associated with MI, with more susceptibility to injury in infants than children. Immura H et al./2001 [55]
					31 ≥ 12 months		
Elective ventricular septal defect repair in children	3–48 months	STH I crystalloid cardioplegia	Anterograde administrations of 25 mL/kg for 4 min, followed by a 2 min repeated dose of 15 mL/kg at 20 to 30 min intervals	4–6 °C	21	cTnI	Blood cardioplegia exerted more beneficial effects in heart preservation and significantly attenuated metabolic stress in ischemic conditions. Caputo M et al./2002 [58]
		4:1 dilution blood/STH I crystalloid cardioplegia			19		
Cardiac surgery	4.5–98 months	STH I crystalloid cardioplegia	Anterograde administrations of induction dose of 110 mL/m^2^/min for 4 min and maintenance dose of 110 mL/m^2^/min for 2 min at 20 to 30 min intervals	4 °C	32	cTnI	Cold blood with warm blood cardioplegic solution was the optimal approach for cyanotic patients. Modi P et al./2004 [59]
		4:1 dilution blood/STH I crystalloid cardioplegia		4 °C	36		
		Cold blood cardioplegia with terminal warm blood cardioplegic reperfusion	Induction and maintenance doses were the same during aorta cross-clamping, and then, the same dose was administered for 2 min at 37 °C immediately before unclamping	4 °C and terminal reperfusion at 37 °C	35		
AV septal defects repair surgery	0–1 year	Plegisol	Anterograde administrations of 20 mL/kg; 10 mL/kg every 20–30 min	4 °C	15 15	CK-MB	Blood cardioplegia preserved myocardial function more effectively than crystalloid. Åmark Ket al/2005 [61]
		4:1 crystalloid/blood					
Arterial switch operation	<30 days	Intermittent warm blood cardioplegia	1–1.5 times the physiological coronary flow rate infused anterogradely for 1 min every 10 min	35–36 °C	188	cTn-I	Better myocardial protection was achieved with repeated oxygenated WBC. Bojan M et al./2013 [65]
		Custodiol	30 mL/min for 7 min	4 °C	30		
Arterial switch operation	<30 days	Blood cardioplegia	5 mL/kg/min, initially for 3 min through ascending aorta and repeated after 20 min	28 °C	44	cTn-I, CK-MB, BNP	Similar extent of myocardial damage and postoperative outcome. Giordano R et al./2016 [67]
		Custodiol	1 mL/min/g of heart weight	5–8 °C	50		
Elective repair of ventricular septal defects and tetralogy of Fallot	≤12 years	4:1 dilution blood/STH I crystalloid cardioplegia	30 mL/kg initially, followed by repeated doses of 15 mL/kg at 25 to 30 min intervals	4 °C	50	cTn-I	del Nido solution exerted more beneficial effects in terms of preservation of cardiac structure, decrease in cTn-I release, and reduced morbidity. Talwar S et al./2017 [68]
		del Nido cardioplegia solution	20 mL/kg single dose was administered through the aortic root		50		
Corrective cardiac surgery	3–69 months	Conventional blood cardioplegia	30 mL/kg dose was repeated beyond an ischemic time of 90 min for del Nido solution. Additionally, the dose was repeated after 20 min for blood cardioplegia	8–12 ° C	30	cTn-I, CK-MB	Both forms of cardioplegia were associated with similar time-related changes in cTn-I and CK-MB, thus suggesting similar myocardial protection. The advantages of del Nido solution involved decreased necessity for inotropic myocardial support and faster recovery of the heart rhythm. Panigrahi D et al./2018 [69]
		del Nido cardioplegia solution			30		
Tetralogy of Fallot	0–18 years	Standard blood cardioplegia	Anterograde administrations of 20 mL/kg, every 20 min, repeated dose of 10 mL/kg	8–12 °C	26	CK-MB	Similar troponin T release was noticed in both groups, thus suggesting myocardial protection was achieved after blood and del Nido cardioplegic solutions. Negi SL et al./2019 [73]
		del Nido cardioplegia solution	20 mL/kg and subsequent dose if cross-clamp time exceeded 75 min	4–8 °C	30		
Surgical repair of congenital heart disease	1–120 months	Blood cardioplegia	30 mL/kg every 4 min	4–6 °C	40	cTn-I	dN cardioplegia enables shorter aortic cross-clamp time and leads to a reduced level of cTn-I. Isildak FU et al./2021 [74]
		del Nido cardioplegia solution	20 mL/kg anterogradely, repeated dose for a procedure longer than 60–90 min		40		
Correction of tetralogy of Fallot	8.3–16.4 months	Modified STH solution	Initially, 30 mL/kg anterogradely and every 40 min at 10 mL/kg	30 °C	27	cTn-I	cTn-I levels were elevated; nevertheless, no significant difference was observed between groups. Gorjipour F et al./2017 [76]
		del Nido cardioplegia solution	Initially, 20 mL/kg and subsequently, 10 mL/kg after 90 min		32		

## 6. Conclusions

In the majority of studies, blood cardioplegia exerted more beneficial effects in pediatric heart preservation compared to crystalloid; nevertheless, it is important to note that the degree of myocardial injury is also strongly affected by the type and duration of the surgical maneuver, overall patient condition, and existence of comorbidities, etc. Hypothermic cardioplegia has been proposed as a strategy that provides the greatest protection for cardiomyocytes during ischemia. Moreover, intraoperative hematocrit changes during open heart surgery represent an important issue to be considered, and blood cardioplegia appears to have an advantage over crystalloid solutions since it is associated with higher hematocrit levels and a reduced risk for postoperative organ failure due to hemodilution. The uniform recommendations among health institutions worldwide do not exist, and so far, the most important determinants for the choice of the myocardial protection method are the clinical results and the surgeons’ experiences. Additionally, the method should be effective, simple, and economically acceptable. An experienced surgeon chooses a cardioplegic solution based on the individual needs of patients in order to provide optimal heart protection while reducing complications.

## Figures and Tables

**Table 1:** Crystalloid cardioplegic solutions.

CRYSTALLOID SOLUTIONS						
Extracellular Solutions				Intracellular Solutions		
Components (mmol/L)	STH 1	Plegisol (STH 2)	Celsior	Custodiol	University of Wisconsin	Eurocollins
Na^+^	144.00	110.00	100.00	15.00	25.00	10.00
K^+^	20.00	16.00	15.00	9.00	120.0	115.00
Mg^2+^	16.00	16.00	13.00	4.00	5.00	/
Ca^2+^	2.40	1.20	0.25	0.015	/	/
Procain hidrochloryde	1.00	/	/	/	/	/
Bicarbonate	/	10.00	/	/	/	10.00
Histidine	/	/	30.00	198.00	/	/
Phosphate buffer	/	/	/	/	25.00	100.00
Mannitol	/	/	60.00	30.00	/	60.00
Glucose	/	/	/	/	/	180.00
Raffinose	/	/	30.00	/	30.00	/
Typtophan	/	/	/	2.00	/	/
Ketoglutarate	/	/	/	1.00	/	/
Adenosine	/	/	/	/	5.00	/
Glutamate	/	/	20.00	/	/	/
Glutathione	/	/	3.00	/	3.00	/
Allopurinol	/	/	/	/	1.00	/
Lactobionate	/	/	80.00	/	100.00	80.00
Osmolarity	320	300	320	300	330	375

**Table 2:** Del Nido solution.

Components	Del Nido Solution
KCl	26 mEq
Bicarbonates	13 mL
Citrate-phosphate-dextrose	/
Mannitol 20%	16 mL
Lidocaine 2%	6.50 mL
Tromethamine 0.3 m	/
Crystalloid:blood ratio	4:1

## Data Availability

Data is contained within the article.

## References

[1] Ghimire A., Bisset E., Howlett S. (2019). Ischemia and reperfusion injury following cardioplegic arrest is attenuated by age and testosterone deficiency in male but not female mice. Biol. Sex Differ..

[2] Tsao P.C., Shiau Y.S., Chiang S.H., Ho H.C., Liu Y.L., Chung Y.F., Lin L.-J., Chen M.R., Chang J.K., Soong W.J. (2016). Development of a Newborn Screening Program for Critical Congenital Heart Disease (CCHD) in Taipei. PLoS ONE.

[3] Chambers D.J., Fallouh H.B. (2010). Cardioplegia and cardiac surgery: Pharmacological arrest and cardioprotection during global ischemia and reperfusion. Pharmacol. Ther..

[4] Glöckner A., Ossmann S., Ginther A., Kang J., Borger M.A., Hoyer A., Dieterlen M.T. (2021). Relevance and Recommendations for the Application of Cardioplegic Solutions in Cardiopulmonary Bypass Surgery in Pigs. Biomedicines.

[5] Drury N.E., Horsburgh A., Bi R., Willetts R.G., Jones T.J. (2019). Cardioplegia practice in paediatric cardiac surgery: A UK & Ireland survey. Perfusion.

[6] Yang X., An N., Zhong C., Guan M., Jiang Y., Li X., Zhang H., Wang L., Ruan Y., Gao Y. (2020). Enhanced cardiomyocyte reactive oxygen species signaling promotes ibrutinib-induced atrial fibrillation. Redox Biol..

[7] Zhu H., Zhou H. (2021). Novel Insight into the Role of Endoplasmic Reticulum Stress in the Pathogenesis of Myocardial Ischemia-Reperfusion Injury. Oxidative Med. Cell. Longev..

[8] Hausenloy D.J., Yellon D.M. (2013). Myocardial ischemia-reperfusion injury: A neglected therapeutic target. J. Clin. Investig..

[9] Dragasevic N., Jakovljevic V., Zivkovic V., Draginic N., Andjic M., Bolevich S., Jovic S. (2021). The role of aldosterone inhibitors in cardiac ischemia-reperfusion injury. Can. J. Physiol. Pharmacol..

[10] Rankovic M., Krivokapic M., Bradic J., Petkovic A., Zivkovic V., Sretenovic J., Jeremic N., Bolevich S., Kartashova M., Jeremic J. (2021). New Insight Into the Cardioprotective Effects of Allium ursinum L. Extract Against Myocardial Ischemia-Reperfusion Injury. Front. Physiol..

[11] Magovern J.A., Pae W.E., Miller C.A., Waldhausen J.A. (1988). The immature and the mature myocardium. Responses to multidose crystalloid cardioplegia. J. Thorac. Cardiovasc. Surg..

[12] Ost’ádal B., Ost’ádalová I., Skárka L., Kolár F., Kopecký J. (2002). Ischemic injury of the developing heart. Exp. Clin. Cardiol..

[13] Doenst T., Schlensak C., Beyersdorf F. (2003). Cardioplegia in pediatric cardiac surgery: Do we believe in magic?. Ann. Thorac. Surg..

[14] Allen B.S. (2004). Pediatric myocardial protection: Where do we stand?. J. Thorac. Cardiovasc. Surg..

[15] Carvajal C., Goyal A., Tadi P. (2022). Cardioplegia.

[16] Gerbode F., Melrose D. (1958). The use of potassium arrest in open cardiac surgery. Am. J. Surg..

[17] Effler D.B., Groves L.K., Sones F.M., Kolff W.J. (1956). Elective cardiac arrest in open-heart surgery; report of three cases. Cleve. Clin. Q..

[18] Francica A., Tonelli F., Rossetti C., Tropea I., Luciani G.B., Faggian G., Dobson G.P., Onorati F. (2021). Cardioplegia between Evolution and Revolution: From Depolarized to Polarized Cardiac Arrest in Adult Cardiac Surgery. J. Clin. Med..

[19] Kirklin J.W., Conti V.R., Blackstone E.H. (1979). Prevention of myocardial damage during cardiac operations. N. Engl. J. Med..

[20] Mentzer R.M., Rahko P.S., Molina-Viamonte V., Canver C.C., Chopra P.S., Love R.B., Cook T.D., Hegge J.O., Lasley R.D. (1997). Safety, tolerance, and efficacy of adenosine as an additive to blood cardioplegia in humans during coronary artery bypass surgery. Am. J. Cardiol..

[21] Tyers G.F., Todd G.J., Niebauer I.M., Manley N.J., Waldhausen J.A. (1975). The mechanism of myocardial damage following potassium citrate (Melrose) cardioplegia. Surgery.

[22] Jynge P., Hearse D.J., Feuvray D., Mahalu W., Canković-Darracott S., O’Brien K., Braimbridge M.V. (1981). The St. Thomas’ hospital cardioplegic solution: A characterization in two species. Scand. J. Thorac. Cardiovasc. Surg. Suppl..

[23] Ledingham S.J., Braimbridge M.V., Hearse D.J. (1987). The St. Thomas’ Hospital cardioplegic solution. A comparison of the efficacy of two formulations. J. Thorac. Cardiovasc. Surg..

[24] Bretschneider H.J. (1980). Myocardial protection. Thorac. Cardiovasc. Surg..

[25] Demmy T.L., Molina J.E., Ward H.B., Gorton M.E., Kouchoukos N.T., Schmaltz R.A., Shennib H. (2008). Custodiol versus Plegisol: A phase 3 multicentre myocardial protection study. Int. J. Angiol..

[26] Reynolds A.C., Asopa S., Modi A., King N. (2021). HTK versus multidose cardioplegias for myocardial protection in adult cardiac surgery: A meta-analysis. J. Card. Surg..

[27] Dobson G.P., Faggian G., Onorati F., Vinten-Johansen J. (2013). Hyperkalemic cardioplegia for adult and pediatric surgery: End of an era?. Front. Physiol..

[28] Sternbergh W.C., Brunsting L.A., Abd-Elfattah A.S., Wechsler A.S. (1989). Basal metabolic energy requirements of polarized and depolarized arrest in rat heart. Am. J. Physiol..

[29] Tyers G.F. (1975). Metabolic arrest of the ischemic heart. Ann. Thorac. Surg..

[30] Oliveira M.A.B., Godoy M.F., Braile D.M., Lima-Oliveira A.P.M. (2005). Polarizing cardioplegic solution: State of the art. Braz. J. Cardiovasc. Surg..

[31] Habertheuer A., Kocher A., Laufer G., Andreas M., Szeto W.Y., Petzelbauer P., Ehrlich M., Wiedemann D.L. (2014). Cardioprotection: A review of current practice in global ischemia and future translational perspective. Biomed. Res. Int..

[32] Gundry S.R., Kirsh M.M. (1984). A comparison of retrograde cardioplegia versus antegrade cardioplegia in the presence of coronary artery obstruction. Ann. Thorac. Surg..

[33] Haan C., Lazar H.L., Bernard S., Rivers S., Zallnick J., Shemin R.J. (1991). Superiority of retrograde cardioplegia after acute coronary occlusion. Ann. Thorac. Surg..

[34] Shirai T., Rao V., Weisel R.D., Ikonomidis J.S., Hayashida N., Ivanov J., Carson S., Mohabeer M.K., Mickle D.A.G. (1996). Antegrade and retrograde cardioplegia: Alternate or simultaneous?. J. Thorac. Cardiovasc. Surg..

[35] Rosenkranz E.R., Vinten-Johansen J., Buckberg G.D., Okamoto F., Edwards H., Bugyi H. (1982). Benefits of normothermic induction of blood cardioplegia in energy-depleted hearts, with maintenance of arrest by multidose cold blood cardioplegic infusions. J. Thorac. Cardiovasc. Surg..

[36] Ahmed A.A., Mahboobi S.K. (2021). Warm Blood Cardioplegia.

[37] Fan Y., Zhang A.M., Xiao Y.B., Lau D.H.H., Morgan K., Magni F., Harky A. (2010). Warm versus cold cardioplegia for heart surgery: A meta-analysis. Eur. J. Cardiothorac. Surg..

[38] Martin T.D., Craver J.M., Gott J.P., Weintraub W.S., Ramsay J., Mora C.T., Guyton R.A. (1994). Prospective, randomized trial of retrograde warm blood cardioplegia: Myocardial benefit and neurologic threat. Ann. Thorac. Surg..

[39] Buckberg G.D. (1994). Normothermic blood cardioplegia. Alternative or adjunct?. J. Thorac. Cardiovasc. Surg..

[40] Hayashi Y., Ohtani M., Sawa Y., Hiraishi T., Akedo H., Kobayashi Y., Nakamura T., Matsuda H. (2005). Initial, continuous and intermittent bolus cardioplegia administration: Efficacy of potassium-chloride and magnesium-sulfate as minimal additives for minimally-diluted blood cardioplegia. J. Cardiovasc. Surg..

[41] Lazar H.L., Rivers S., Cambrils M., Bernard S., Shemin R.J. (1991). Continuous versus intermittent cardioplegia in the presence of a coronary occlusion. Ann. Thorac. Surg..

[42] Gunnes S., Jynge P., Podesser B.K., Chambers D.J. (2011). Fundamentals of the Past: Cardioplegia: The First Period Revisited. New Solutions for the Heart.

[43] Singh S., De D., Spadaccio C., Berry C., Al-Attar N. (2017). An overview of different methods of myocardial protection currently employed peri-transplantation. Vessel. Plus.

[44] Sanetra K., Pawlak I., Cisowski M. (2018). Del Nido cardioplegia–what is the current evidence?. Kardiochir Torakochirurgia Pol..

[45] El-Morsy G.Z., Abdullah H.M., Abo-Haded H.M., Elgamal M.A.F., El-Deep A.M. (2014). Does type of cardioplegia affect myocardial and cerebral outcome in pediatric open cardiac surgeries?. Ain-Shams J. Anesthesiol..

[46] Chen Y., Shi J., Xia T.C., Xu R., He X., Xia Y. (2019). Preservation Solutions for Kidney Transplantation: History, Advances and Mechanisms. Cell Transplant..

[47] Aarsaether E., Stenberg T.A., Jakobsen Ø., Busund R. (2009). Mechanoenergetic function and troponin T release following cardioplegic arrest induced by St Thomas’ and histidine-tryptophan-ketoglutarate cardioplegia--an experimental comparative study in pigs. Interact. Cardiovasc. Thorac. Surg..

[48] Rosenkranz E.R., Buckberg G.D. (1983). Myocardial protection during surgical coronary reperfusion. J. Am. Coll. Cardiol..

[49] Follette D.M., Mulder D.G., Maloney J.V., Buckberg G. (1978). Advantages of blood cardioplegia over continuous coronary perfusion or intermittent ischemia. Experimental and clinical study. J. Thorac. Cardiovasc. Surg..

[50] Shiroishi M.S. (1999). Myocardial protection: The rebirth of potassium-based cardioplegia. Tex. Heart Inst. J..

[51] Øvrum E., Tangen G., Tølløfsrud S., Øystese R., Ringdal M.A., Istad R. (2010). Cold blood versus cold crystalloid cardioplegia: A prospective randomised study of 345 aortic valve patients. Eur. J. Cardiothorac. Surg..

[52] Kim K., Ball C., Grady P., Mick S. (2014). Use of del Nido Cardioplegia for Adult Cardiac Surgery at the Cleveland Clinic: Perfusion Implications. J. Extra Corpor. Technol..

[53] Matte G.S., del Nido P.J. (2012). History and use of del Nido cardioplegia solution at Boston Children’s Hospital. J. Extra Corpor. Technol..

[54] Young J.N., Choy I.O., Silva N.K., Obayashi D.Y., Barkan H.E. (1997). Antegrade cold blood cardioplegia is not demonstrably advantageous over cold crystalloid cardioplegia in surgery for congenital heart disease. J. Thorac. Cardiovasc. Surg..

[55] Imura H., Caputo M., Parry A., Pawade A., Angelini G.D., Suleiman M.S. (2001). Age-dependent and hypoxia-related differences in myocardial protection during pediatric open heart surgery. Circulation.

[56] Cyran S.E., Phillips J., Ditty S., Baylen B.G., Cheung J., LaNoue K. (1993). Developmental differences in cardiac myocyte calcium homeostasis after steady-state potassium depolarization: Mechanisms and implications for cardioplegia. J. Pediatr..

[57] Wittnich C., Peniston C., Ianuzzo D., Abel J.G., Salerno T.A. (1987). Relative vulnerability of neonatal and adult hearts to ischemic injury. Circulation.

[58] Caputo M., Modi P., Imura H., Pawade A., Parry A.J., Suleiman M.S., Angelini G.D. (2002). Cold blood versus cold crystalloid cardioplegia for repair of ventricular septal defects in pediatric heart surgery: A randomized controlled trial. Ann. Thorac. Surg..

[59] Modi P., Suleiman M.S., Reeves B., Pawade A., Parry A.J., Angelini G.D., Caputo M. (2004). Myocardial metabolic changes during pediatric cardiac surgery: A randomized study of 3 cardioplegic techniques. J. Thorac. Cardiovasc. Surg..

[60] Hammon J.W., Graham T.P., Boucek R.J., Parrish M.D., Merrill W.H., Bender H.W. (1987). Myocardial adenosine triphosphate content as a measure of metabolic and functional myocardial protection in children undergoing cardiac operation. Ann. Thorac. Surg..

[61] Amark K., Berggren H., Björk K., Ekroth A., Ekroth R., Nilsson K., Sunnegårdh J. (2005). Blood cardioplegia provides superior protection in infant cardiac surgery. Ann. Thorac. Surg..

[62] Sinha P., Zurakowski D., Jonas R.A. (2008). Comparison of two cardioplegia solutions using thermodilution cardiac output in neonates and infants. Ann. Thorac. Surg..

[63] Charette K., Gerrah R., Quaegebeur J., Chen J., Riley D., Mongero L., Corda R., Bacha E. (2012). Single dose myocardial protection technique utilizing del Nido cardioplegia solution during congenital heart surgery procedures. Perfusion.

[64] Durandy Y.D., Younes M., Mahut B. (2008). Pediatric warm open heart surgery and prolonged cross-clamp time. Ann. Thorac. Surg..

[65] Bojan M., Peperstraete H., Lilot M., Tourneur L., Vouhé P., Pouard P. (2013). Cold histidine-tryptophan-ketoglutarate solution and repeated oxygenated warm blood cardioplegia in neonates with arterial switch operation. Ann. Thorac. Surg..

[66] Qulisy E., Fakiha A., Debis R., Jamjoom A.A., Elassalac A.A., Al-Radi O. (2016). Custodiol versus blood cardioplegia in pediatric cardiac surgery, two-center study. J. Egypt. Society Cardio-Thoracic Surg..

[67] Giordano R., Arcieri L., Cantinotti M., Pak V., Poli V., Maizza A., Melo M., Assanta N., Moschetti R., Murzi B. (2016). Custodiol Solution and Cold Blood Cardioplegia in Arterial Switch Operation: Retrospective Analysis in a Single Center. Thorac. Cardiovasc. Surg..

[68] Talwar S., Bhoje A., Sreenivas V., Makhija N., Aarav S., Choudhary S.K., Airan B. (2017). Comparison of del Nido and St Thomas Cardioplegia Solutions in Pediatric Patients: A Prospective Randomized Clinical Trial. Semin. Thorac. Cardiovasc. Surg..

[69] Panigrahi D., Roychowdhury S., Guhabiswas R. (2018). Myocardial protection following del Nido cardioplegia in pediatric cardiac surgery. Asian Cardiovasc. Thorac. Ann..

[70] O’Brien J.D., Howlett S.E., Burton H.J., O’Blenes S.B., Litz D.S., Friesen C.L. (2009). Pediatric cardioplegia strategy results in enhanced calcium metabolism and lower serum troponin T. Ann. Thorac. Surg..

[71] Snabaitis A.K., Shattock M.J., Chambers D.J. (1997). Comparison of polarized and depolarized arrest in the isolated rat heart for long-term preservation. Circulation.

[72] Yang Y.J. (1991). Protection of immature myocardium by the addition of mannitol to crystalloid cardioplegic solution. J. Formos. Med. Assoc..

[73] Negi S.L., Mandal B., Singh R.S., Puri G.D. (2019). Myocardial protection and clinical outcomes in Tetralogy of Fallot patients undergoing intracardiac repair: A randomized study of two cardioplegic techniques. Perfusion.

[74] Isildak F.U., Yavuz Y. (2021). Comparison of Del Nido and Blood Cardioplegia in Pediatric Patients Undergoing Surgical Repair for Congenital Heart Disease. Pediatr. Cardiol..

[75] Mohammed S., Menon S., Gadhinglajkar S.V., Baruah S.D., Ramanan S.V., Gopalakrishnan K.A., Suneel P.R., Dharan B.S. (2022). Clinical outcomes of del nido cardioplegia and st thomas blood cardioplegia in neonatal congenital heart surgery. Ann. Card. Anaesth..

[76] Gorjipour F., Dehaki M.G., Totonchi Z., Hajimiresmaiel S.J., Azarfarin R., Pazoki-Toroudi H., Mahdavi M., Korbi M., Dehaki M.G., Soltani B. (2017). Inflammatory cytokine response and cardiac troponin I changes in cardiopulmonary bypass using two cardioplegia solutions; del Nido and modified St. Thomas’: A randomized controlled trial. Perfusion.

[77] Stammers A.H., Tesdahl E.A. (2017). Does the Type of Cardioplegic Technique Influence Hemodilution and Transfusion Requirements in Adult Patients Undergoing Cardiac Surgery?. J. Extra Corpor. Technol..

[78] Günday M., Bingöl H. (2014). Is crystalloid cardioplegia a strong predictor of intra-operative hemodilution?. J. Cardiothorac. Surg..

